# The Effect of Active Creation on Psychological Health: A Feasibility Study on (Therapeutic) Mechanisms

**DOI:** 10.3390/bs8020025

**Published:** 2018-02-12

**Authors:** Gudrun Lange, Rainer Leonhart, Harald Gruber, Sabine C. Koch

**Affiliations:** 1 Therapeutic Sciences, Dance Movement Therapy, SRH University Heidelberg, 69123 Heidelberg, Germany; sabine.koch@alanus.edu; 2 Department of Psychology, University of Freiburg, 79085 Freiburg, Germany; leonhart@psychologie.uni-freiburg.de; 3 Research Institute of Creative Arts Therapies (RIArT), Alanus University, 53347 Alfter, Germany; harald.gruber@alanus.edu

**Keywords:** active creation, working factors, self-efficacy, well-being, mediation analysis, artistic inquiry, mixed methods, embodied aesthetics, creative arts therapies

## Abstract

Creation is an important part of many interventions in creative arts therapies (art, music, dance, and drama therapy). This active part of art-making in arts therapies has not yet been closely investigated. The present study commits to this field of research using a mixed-methods design to investigate the effects of active creation on health-related psychological outcomes. In an artistic inquiry within an experimental design, *N* = 44 participants engaged in active art-making for eight minutes in the presence of the researcher (first author) with a choice of artistic materials: paper and colors for drawing and writing, musical instruments, space for moving or performing. Before and after the creation, participants completed a well-being, a self-efficacy and an experience of creation scale, and in addition found their own words to express the experiences during the activity. We hypothesized that the experience of empowerment, freedom, impact, and creativity (Experience of Creation Scale) mediates the positive effect of active creation on the outcomes of self-efficacy and well-being, and evaluated this assumption with a mediation analysis. Results suggest that the effect of active creation on both self-efficacy and well-being is significantly mediated by the Experience of Creation Scale. This article focuses on the quantitative side of the investigation. During the process, qualitative and quantitative results were triangulated for a more valid evaluation and jointly contribute to the emerging theory frame of embodied aesthetics.

## 1. Introduction

Creative arts therapies (CATs) are applied in clinical and psychiatric settings and increasingly report positive health-related outcomes [1]. Despite the effective practice, scientific research is needed in order to understand exactly how CATs work [2]. This understanding is relevant not only within therapeutic work, but also for the positioning of CATs in the health sector [3].

**Figure 1:**
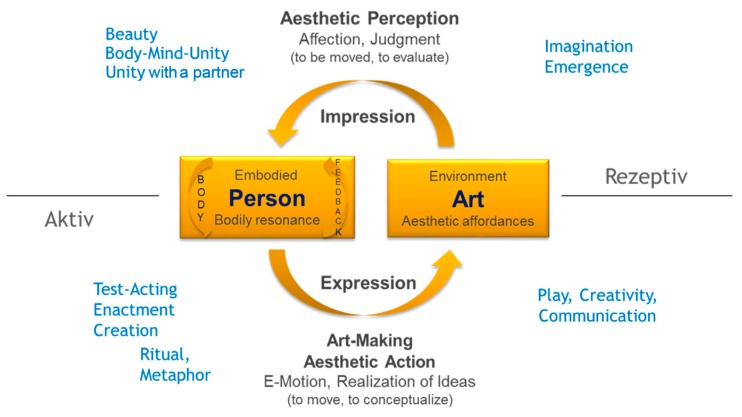
The Model of Embodied Aesthetics with loosely assigned therapeutic factors [2] (p. 89).

Trudi Schoop, a pioneer of dance movement therapy, acknowledges creation as a main element in her therapeutic work [4]. Similarly, creation has been described as crucial in the different creative arts therapies’ disciplines [5,6,7,8]. Based on Schoop’s definition, the term creation is understood in this study as an autonomous process of shaping, in which all decisions are bound to the creating person. In order to frame creation as a therapeutic intervention theoretically, we employed the Model of Embodied Aesthetics [2] (p. 87), Figure 1. 

In the model, the active and receptive side of art-making are conceptualized in a circular manner, with expression and impression forming a unity. Since cognitive science models lack the conceptualization of the active side of art-making [2], the model of embodied aesthetics is necessary to represent the main workings of arts therapies and ground the respective therapeutic working factors. Creation as an aesthetic process is located on the active side. This research focuses on this only recently modelled active side (expression, art making; see lower part of Figure 1) in order to understand more closely its factors at work.

There are publications about the therapeutic factors in different CATs: Hillecke and Wilker (2007) develop a model for music therapy, Koch and Eberhard-Kaechele describe therapeutic factors for dance movement therapy, and Oepen does so for art therapy [9,10,11]. Imus describes the creative process as a general criterion in all CATs and emphasizes that in the different disciplines the creative process is utilized in a similar way [12]. In the present study, we examined the act of creating and its implications for the health-related psychological outcomes of well-being and self-efficacy. Our focus lies on the active and the creative across all creative arts therapies, not the specificity of each artistic discipline. Koch hypothesised therapeutic factors of CATs bound to the Model of Embodied Aesthetics, but there are only very few empirical studies about these factors of CATs [2] (p. 89). This study focuses on the overarching effect of creation across arts modalities in one single intermodal intervention and asks: Does creation positively affect our psychological health?

Following the WHO’s multidimensional definition of health from 1948 we understand health as physical, psychological and social well-being [13]. In this study, we focused on the health-related psychological outcomes of well-being, as conceptualized by Goodill [14] and operationalized by Koch and colleagues [15,16], and of self-efficacy, as Bandura conceptualized it as fundamental for human health [17], and Schwarzer and Jerusalem operationalized it [18] in the General Self Efficacy-scale (GSE). 

Based on working 18 years in the arts creating dances and performances together with both professionals and amateurs, the first author (GL) constructed a scale of four experience- and observation-based items to be tested in the context of the following research question: Does the experience of empowerment, freedom, impact, and creativity mediate the positive effect of active creation on human health?

## 2. Materials and Methods

**Figure 2:**
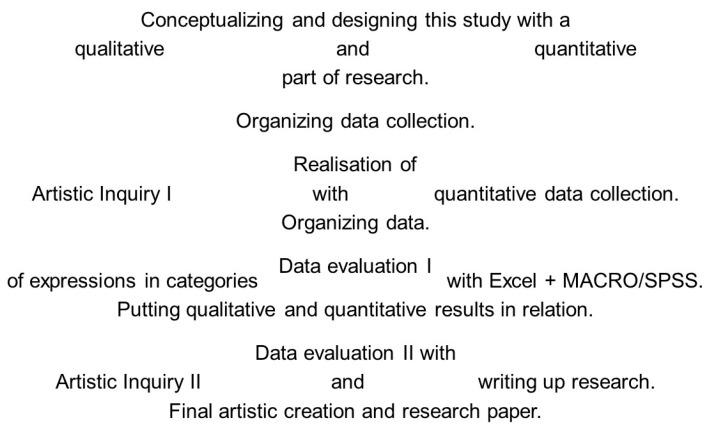
Process model of the study.

The study was set up as a mixed-methods design as described in Figure 2. We used a concurrent combination of two state-of-the-art methods for analyzing questions on working factors/mechanisms: mediation analysis [19], as recommended by the National Institute of Health (NIH) of the USA for analysis of therapeutic mechanisms in the (integrative) health sciences, and artistic inquiry as a state-of-the-art method development unique to the arts therapies. Within an experimental frame, artistic inquiry was used for qualitative data collection and analysis [20], and mediation analysis for quantitative data analysis [19]. A triangulation approach was implemented to let the methods inform each other. The researchers take a humanistic clinical stance and a transformational epistemological stance (compare Creswell & Creswell [21]; Mertens & Hesse-Biber [22]).

### 2.1. Sample

Forty-four participants (7 men, 37 women) took part in the study at SRH University, Germany. The sample consisted of students of dance movement therapy, music therapy, and occupational therapy, with a mean age of 25.98 years (standard deviation: SD = 6.67, age range: 19–52). They were divided into an experimental group (EG, *N* = 21; 19 women and 2 men) and a control group (CG, *N* = 23; 18 women and 5 men). See Table 1 for details on demographic data. Power estimations indicate that in a model with one mediator, *N* = 44 participants reach a power of 0.81 (with an aspired power of 0.80 in this feasibility study), assuming correlations of *r* = 0.7 between the independent variable *X*, the dependent variable *Y*, and the mediator *M* (estimated with the online-tool by Schoemann and colleagues [23]: https://schoemanna.shinyapps.io/mc_power_med/). 

We announced the experiment, using students’ mailing lists of SRH University, and went into students’ courses and addressed students directly on-site at SRH campus during their breaks. A mailing list was created, and a doodle calendar with an invitation to participate was sent out. We announced that the study takes a closer look at creating, that they should have previous experience with artistic media, that the participants would need to improvise alone in an individual setting choosing movement, music, colors, and/or words, that the researcher would answer in an improvisation, and that there would be questionnaires to complete. We advertised that we would provide chocolate bars and course credit (certificates of participation hours needed by students) as a reward. In order to recruit as many participants as possible, students could inscribe using a doodle calendar. The sample was thus not randomized, but *t*-test and Chi-square test yielded that there were no significant baseline differences between EG and CG (all *p*-values > 0.279). We identified three systematic outliers in the EG for the outcome of well-being and excluded them in the according analyses. Thus, 41 participants (19 EG, 22 CG) remained in the analysis of well-being, and 44 participants (22 EG, 22 CG) remained in the analysis of self-efficacy. The CG participants participated in one of two regular classes (either on theories and techniques of supervision or conceptualization of health and sickness) completing the identical questionnaires at the beginning and in the end of the class. They were contacted through their professor, and the researcher (first author) was present before the start of their classes to raise attention for the study and clarify questions. 

### 2.2. Procedure

**Figure 3:**
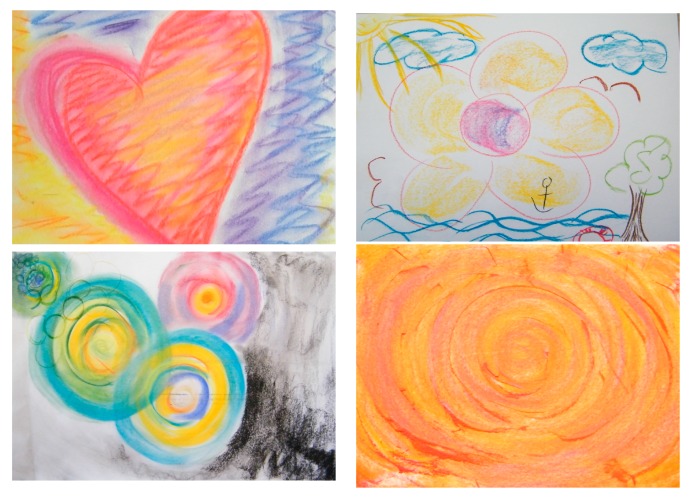
These photos show the first four examples/details of the creations with paper, color, and/or words that emerged from the artistic inquiry in this study.

**Figure 4:**
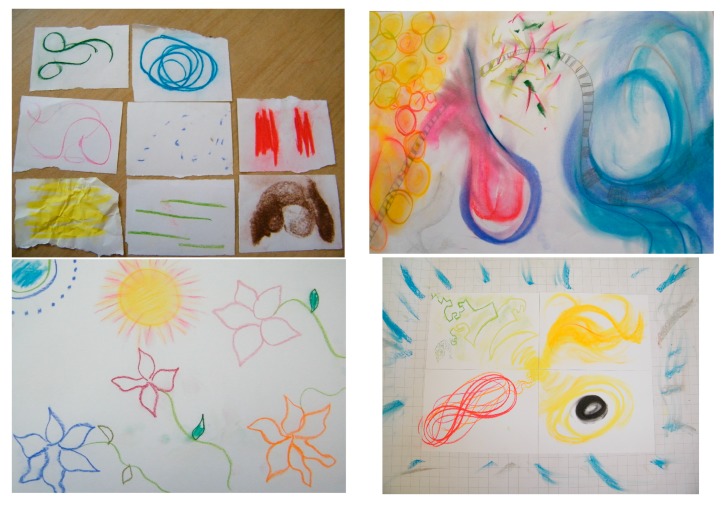
Photos of the second four creations with paper, color, and/or words that emerged from the artistic inquiry in this study.

The individual settings were conducted in a 75 m^2^-music therapy room at SRH University Heidelberg. It was equipped with mats, a lounge area, materials for visual arts, and 62 instruments. Two video cameras and an audio recorder were prepared (for exact lists for replication purposes, please contact first author.) EG participants were welcomed. If they did not know the space, they were invited to look around and get acquainted with the environment. Then, the participant was asked to sit down with the researcher in the lounge area in order to be introduced to the study. The participant signed the informed consent sheet and filled out the first questionnaire (t1) after receiving information about coding. Then the researcher gave a short introduction, asked her counterpart what the term “Gestalten” (creating) means for him or her and ended with a description of what to expect following a protocol (please contact the first author for German original instructions.). Participants were asked to improvise with movement, music, colors and/or words for a maximum of eight min. They were allowed to use any media and could also stop earlier (see Figure 3, Figure 4, Figures 7 and 8 for illustrating examples of resulting visual art creations). Timing was controlled, movement videotaped, and music recorded. The opening question for the improvisation was: What happens inside yourself when you create?

Afterward, participants and researcher (GL) independently phrased an expression in written words about what they just had experienced followed by a verbal exchange. A questionnaire was then filled in for the second time (t2). As a further step, the researcher improvised in reaction to the participant’s improvisation under the same conditions (8 min; all media). Again, participant and researcher phrased an expression capturing their experience, had a verbal exchange, and the questionnaire was then completed for the last time (t3). Ending the session, participants could choose from a choice of chocolate bars. We concurrently provided the certificates of participation hours, debriefed the participants, and thanked them for attending. One session took about 45 to 50 min. CG participants followed regular class without artistic creating and completed two questionnaires at t1 and t2. These two measurement points in EG and CG, served in the quantitative analyses reported here.

### 2.3. Materials and Instruments

The room was prepared with four pieces of paper. At each wall one of them was attached reading either “Movement” or “Music” or “Words” or “Colors/Forms”. The offered musical instruments were a timbale, a drum set, a kettledrum, 5 congas, 3 djembés, 2 tambourines, a vibraphone, a marimbaphone, a xylophone, a glockenspiel, 6 boomwhackers, 6 chimes, a metallophone, a bass chime, a tomtom, a tympanum the size of a table, a bigbom, 4 claves, a temple block, a pair of maracas, a cabaza, 2 caxixis, a cajon, a triangle, an agogobell, a gong, a singing bowle, a thumb piano, an ocean drum, a kazoo, a rain maker, a piano, a guitar, a bowed psaltery, a lyre, a monochord, an accordion, an aerophone, a soprano recorder, an alto recorder, and a slide whistle. The offered visual arts materials were normal paper in A3, thicker paper in A4, 3 pens, 28 wax oil crayons, 12 water color pencils, 24 pastels, different qualities pencils with eraser, and a sharpener. Technical devices were a Zoom H2n audio recorder, a canon camcorder (Legria HF20) (Uxbridge, UK), and a Fujifilm camera (Finepix S1800) (Minato-ku, Tokyo, Japan).

Well-being was measured with the Heidelberg State Inventory (HSI; [15,16]; see Appendix A). The six dimensions of positive affect, coping, fear, depressive affect, tension, and vitality were assessed each with four items. Two items were phrased in correspondence with the positive pole of the dimension, two items were inverted. A vitality-item was “Right now I feel full of energy”. An inverted fear-item was “Right now I feel without sorrow”. These items are to be rated on a scale where “1” means “very low well-being” and “5” “very high well-being”. In this study, the *HSI* showed a high internal consistency of Cronbach’s α = 0.95.

Self-efficacy was measured with the General Self-Efficacy Scale (GSE; [18,24]). Sample items are “When I am confronted with a problem, I can usually find several solutions. “or “If I am in trouble, I can usually think of a solution”. Ten items are rated on a scale from “1” ”not at all true” to “4” “exactly true”. Internal consistencies in international studies resulted in Cronbach’s α = 0.86 in a sample of *N* = 19.120 [25], the same value resulted in this study.

For this study, the Experience of Creation Scale (*ECS*; [26]) has been designed consisting of four items (see Appendix B). These items are “Right now I feel empowered”, “Right now I feel free to decide”, “Right now I feel I have impact”, and “Right now I feel creative” (translated by first author). Participants rated these items on a scale from “1” meaning “not at all true” to “5” meaning “exactly true”. Its internal consistency was Cronbach’s α = 0.89.

The opening question at the start of the improvisation was: What happens inside yourself when you create? The reflective question after creation was: What did you experience while creating? In both cases, the active person had approximately two minutes to write an answer. Resulting statements were collected.

### 2.4. Statistical Analysis

The new measure *ECS* was tested with factor analysis and reliability analysis. For between-group effects, we computed a repeated measures ANOVA using the Greenhouse-Geisser-value controlling for problems with sphericity. The alpha-level was set at 0.05. To determine correlations between scales, we used Pearson’s correlation coefficient. Mediation analysis [19] was conducted in order to determine the effect of *ECS*. Mediation analysis is based on regression analysis. Both the indirect and direct effect of the independent variable (*X*) on the outcome variable (*Y*) are being reported and interpreted. The indirect effect *ab* is the product of the regression coefficients of the regression of *X* on the mediator variable (*M*) *a* and the regression of *M* on *Y b*. The direct effect is called *c′*. For mediation analysis, we conducted multiple linear regression analyses with the Macro PROCESS for SPSS 22 (IBM Corporation, Armonk, NY, USA) from Hayes [19]. For all effects, we report unstandardized regression coefficients [19]. We used bias-corrected boot-strapping obtained with 10,000 resamples [19].

### 2.5. Mixed Methods Analysis

On the background of a transformational epistemological framework [22], data transformation [21] was used as a validation strategy on multiple levels, transforming qualitative into quantitative data; transforming qualitative verbal data into categories, sequencing them and feeding them back into the theory model; transforming qualitative artistic data into different artistic and verbal modalities; and synergizing all data into one final performance of the first author (GL) as an artistic result of the analysis (see [27]) following the leading questions: What is the essence of each creation? How can they be combined into an entity, accommodating each single mode of expression?

## 3. Results

### 3.1. Descriptive Statistics

Mean values and standard deviations for both groups at pre- and post-test are provided in Table 2.

### 3.2. Inferential Statistics

#### 3.2.1. Analysis of Variance

The repeated measures ANOVA (including t1 and t2) yielded a significant difference between pretest and posttest with a multivariate effect for time * condition of *F* = 15.48, *p* = 0.000, eta_p_^2^ = 0.54, and with the according univariate effects (between-group differences) of *F*(1,44) = 13.73, *p* < 0.001, eta_p_^2^ = 0.25 for well-being (*HSI*), *F*(1,44) = 17.99, *p* = 0.000, eta_p_^2^ = 0.46 for self-efficacy (*GSE*), and *F*(1,44) = 35.39, *p* = 0.000, eta_p_^2^ = 0.30 for experienced creation (*ECS*). We computed the Greenhouse-Geisser-values controlling for problems with sphericity [28].

#### 3.2.2. Correlations between Scales

With Pearson’s correlation coefficient the relations between the implemented scales were analyzed (see Table 3). Noticeable is the strong relation between the variables *HSI* and *GSE* at t2 and the theorized mediator *ECS*: *HSI* (t2) correlates significantly with *ECS* (t2) with *r*(39) = 0.69, *p* < 0.001. Also *GSE* (t2) correlates significantly with *ECS* (t2) with *r*(39) = 0.65, *p* < 0.001. 

#### 3.2.3. Reliability of the New Measure ECS

The factor analysis of *ECS* shows one general factor, that explains 74.66% of the variance. Internal consistency is high with Cronbach’s Alpha α = 0.89. Item reduction would not increase Cronbach’s Alpha α as can be seen in Table 4. Based on these findings *ECS* is used as mediator in the following mediation analysis.

#### 3.2.4. Mediation Analyses

**Figure 5:**
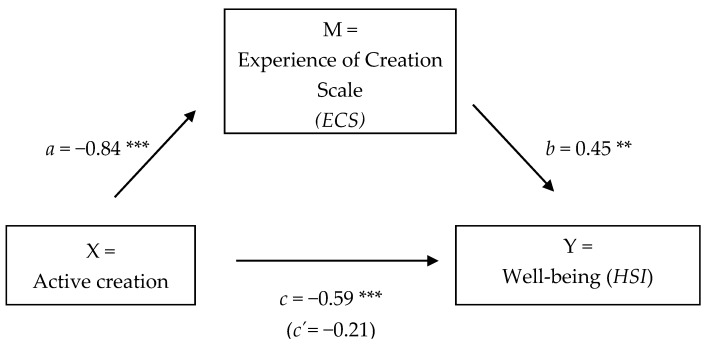
The effect of active creation on well-being (*HSI*) was mediated by the Experience of Creation Scale (*ECS*; full mediation). Note: * *p* < 0.05, ** *p* < 0.01, *** *p* < 0.001, *HSI* = Heidelberg State Inventory [16].

We conducted multiple regression analyses for the outcomes well-being (*HSI*) and self-efficacy (*GSE*) at t2, with the mediator of the experience of active creation measured with the *ECS*, also at t2 (i.e., we used the data collected at posttest t2 to assess each component of the proposed mediation model). For the outcome *HSI* we found that active creation was positively associated with well-being (*HSI*; the total effect *c: B* = −0.59, *t*(39) = −3.58, *p* < 0.001). We also found that active creation was positively related to *ECS* (regression coefficient *a: B* = −0.84, *t*(39)= −4.51, *p* < 0.001). Finally, results indicated that the mediator *ECS* was positively associated with well-being (*HSI*; regression coefficient *b: B*= 0.45, *t*(39)= 3.67, *p* < 0.001). Results are presented in Table 5. Because both, paths a and b were significant, we used mediation analyses with the boot-strapping method with bias-corrected confidence estimates: *B* = −0.38, CI = −0.70; −0.16. Results of the mediation analysis confirmed the mediating role of *ECS* in the relation of active creation and well-being. The 95% confidence interval of the indirect effect was obtained with 10,000 bootstrap resamples [19]. In addition, results indicated that the direct effect *c′* of active creating on well-being (*HSI*) ceased to be significant (*B* = −0.21, *t*(39) = −1.19, *p* = 0.24)*.* Thus, total mediation is suggested. Figure 5 displays the results. 

**Figure 6:**
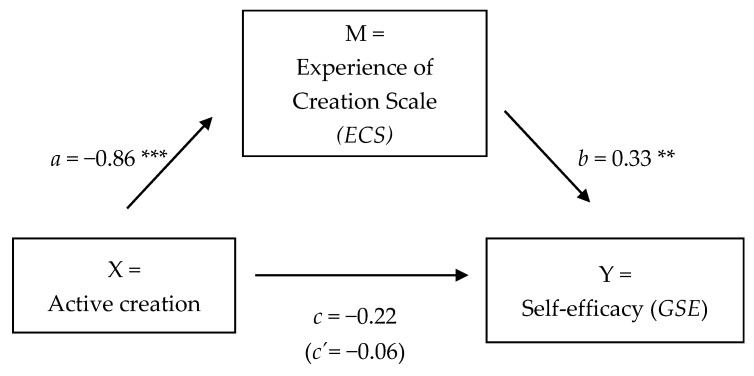
The effect of active creation on self-efficacy (*GSE*), mediated by the Experience of Creation Scale (*ECS*; full mediation)*.* Note: * *p* < 0.05, ** *p* < 0.01, *** *p* < 0.001, *GSE =* General Self-Efficacy Scale [24].

For the outcome self-efficacy (*GSE*), multiple regression analysis was conducted to assess each component, presented in Table 6. First, it was found that active creation was positively associated with self-efficacy (*GSE*; the total effect *c*: *B* = −0.23, *t*(42) = −1.85, *p* = 0.07). It was also found that active creation was positively related to *ECS* (regression coefficient *a*: *B* = −0.85, *t*(42) = −4.87, *p* < 0.001). Finally, results indicated that the mediator *ECS* was positively associated with self-efficacy (*GSE*; regression coefficient *b*: *B* = 0.33, *t*(42) = 3.46, *p* = 0.001). Because both paths a and b were significant, mediation analyses were tested using the bootstrapping method with bias-corrected confidence estimates: *B* = −0.28, CI = −0.53; −0.12. Results of the mediation analysis confirmed the mediating role of *ECS* in the relation between active creating and self-efficacy. The 95% confidence interval of the indirect effect was obtained with 10,000 bootstrap resamples [19]. In addition, results indicated that the direct effect *c*′ of active creating on self-efficacy (*GSE*) ceased to be non-significant (*B* = 0.06, *t*(42) = 0.41, *p* = 0.68). For the outcome of self-efficacy (*GSE*), full mediation was suggested. Figure 6 displays the results. 

#### 3.2.5. Qualitative Results

Due to the richness of the material, only the qualitative results from the open questions are reported here. The resulting data consists of 58 self-phrased expressions that capture the active person’s self-reported experience during creating (translated by first author): 1. Approaching. Searching for the essence. Exploring the essence; 2. Comprehending; 3. “The moment”; 4. TO EXPERIENCE; 5. Relief; 6. It grew and grew; 7. Keen on experimenting; 8. The need to explore; 9. color = shape = sound = dynamics?; 10. colors > tones, rhythm > grey/white, dynamics > shape; 11. Free; 12. Freedom; 13. Joy; 14. Joy, the felicitous variation, in liaison with the search for harmony, dynamic, and the bravery, to strike a new path; 15. Thoughts; 16. Thoughtlessly; 17. Being drawn; 18. Heart-warming; 19. From being in the center of sound to becoming the center of sound; 20. Listening into my spine; 21. It is quiet inside—the outside is gaining momentum; 22. Is one pole stronger than the other or not? 23. Childhood; 24. Bud; 25. Physical rapprochement to remembered sounds; 26. Creative; 27. Creativity; 28. Life; 29. Emptiness; 30. Emptiness and fullness; 31. Singing songs; 32. To entwine, to flourish, to open up, and to close again over night; 33. Rhythm. Sound. Abrupt changes; 34. To peel/strip off, to be with myself and letting it go; 35. Nice feeling; 36. Self-confident; 37. Self-care; 38. To steady oneself; 39. To find voice; 40. Flow of life; 41. Trance; 42. Dreams; 43. To overlay, to wind around each other, to merge, to resist; 44. Convertibility; 45. Conjunction of shapes transformed in space, sound and game; 46. Immersed; 47. Diversity of disruption; 48. From the inside to the outside with power, impact, energy, and the right reasoning on my side; 49. From myself to the outside world and back; 50. Pleasant anticipation; 51. Stepping ahead without cease, following the moment without stress; 52. Warmth; 53. When the (my) eye opens up; 54. Whirling in colors and drumming; 55. Where do these songs come from? 56. TIME; 57. To become aware of myself; 58. Connections.

German original words: Annähernd. Den Punkt suchen. Den Punkt erforschen. · Begreifend · “Der Moment” · ERLEBEN · Erleichterung · Es wurde immer größer. · experimentierfreudig · Explorationsbedürfnis · Farbe = Form = Sound = Dynamik? · Farben > Töne, Rhythmus > grau/weiß, Dynamik > Form · Frei · Freiheit · Freude · Freude, die gelungene Variation, in Verbindung mit der Suche nach Einklang, Dynamik und dem Mut, neue Wege zu beschreiten. · Gedanken · Gedankenlos · > gezogen werden · herzerwärmend · Im Zentrum des Sounds zum Zentrum des Sounds. · in meine Wirbelsäule hineinhorchen. · innen ruhig - außen nimmt es Fahrt auf. · Ist ein Pol stärker als der andere oder nicht? · Kindheit · Knospe · >> körperliche Annäherung an erinnerte Töne << · Kreativ · Kreativität · Leben · Leere · Leere & Fülle · Lieder singen · ranken, aufblühen, öffnen und über Nacht wieder schließen · Rhythmus, Sound. Jäher Wechsel. · Schälen, sein können und weiterziehen lassen · schönes Gefühl · Selbstbewusst · Selbstfürsorge · > sich festigen · Stimme finden · Strom des Lebens · Trance · Träume · Überlagern, umeinander herumschlingen, ineinander aufgehen, widersetzen · Veränderbarkeit · Verknüpfung von/ Formen übersetzt in Raum, Ton & Spiel · Vertieft · Vielfältigkeit der Zerrissenheit · Von innen nach außen mit Kraft, Wucht, Energie und allen guten Argumenten auf meiner Seite · Von mir zur Außenwelt und wieder zurück. · Vorfreude · Vorwärts schreiten ohne Unterlass, ohne Stress dem Moment folgen. · Wärme · Wenn sich das (mein) Auge öffnet… Wirbel in Farbe & im Trommeln · Woher kommen diese Lieder? · ZEIT · zu mir kommen · Zusammenhänge

Resulting statements from the open questions were categorized by a female rater into topics or themes. She was an independent outside rater proficient in artistic inquiry, who in a content analytic densification, summed these expressions in six categories and labelled them (translation by first author): 1. Only me; 2. An impulse hits me/permeates me—I encounter myself; 3. Between outside and inside; 4. From inside to the outside; 5. Become quiet, listen carefully, watch, marvel; 6. A change.

German original words: nur ich · ein Impuls stößt auf mich/durch mich hindurch–ich stoße auf mich · zwischen außen und innen · und von innen nach außen · still sein, zuhören, sehen, staunen · eine Veränderung.

Sixteen expressions were related to the four hypothesized factors empowerment, freedom, impact, and creativity. Forty-two expressions captured various processes that cannot be related to these factors. In addition, there were 38 improvisations in music, movement, colors and/or words that were captured in different media. In the frame of this research, these improvisations were reduced to their essentials by data transformation and were then composed to a final creation, a one-woman (GL) multimedia performance of six minutes duration, using projection of artworks of the participants, music of participants, and movement performance and words generated by participants that related to and counterweighed the quantitative results. Further details on the qualitative results are reported in Lange and Koch [27].

## 4. Discussion

Focusing on the quantitative side of the study, the results of the mediation analysis suggests that experiencing empowerment, freedom, impact, and creativity could be the mechanism increasing the psychological health-outcomes of well-being and self-efficacy, when a person actively creates. Because of the feasibility character of the study, the results are suggestive, not conclusive. While the results are promising, further studies with bigger samples need to follow.

Koch points out that “it is not easy to separate the therapeutic factors into distinct categories, because many of these causes and consequences of art are linked together in processes that intertwine” [2] (p. 90). How about the distinction in this study? We hypothesized that the experience of empowerment, freedom, agency, and creativity while creating has a positive effect on well-being and self-efficacy. Are the concepts of mediator and outcome variable distinguishable categories? One can argue that in order to feel well/healthy, human beings need the experience of the qualities named above. With the concept of self-efficacy, the danger of redundancy is even bigger: Is not the experience of empowerment and impact the experience of self-efficacy itself? This study’s results can help with these questions. Looking at the reliability analysis of *ECS* it becomes apparent that the highest score of Cronbach’s Alpha for *ECS* is the version with all four factors as presented in Table 4. Results can be interpreted the way that a CATs intervention such as creation is accompanied by a cluster of working factors that mediate the effect of the intervention. We hypothesize that this cluster is specific for each intervention. In this perspective, the experience of empowerment and impact is not redundant, but a defining part of creation and creating in the context of CATs.

Qualitative results show how several transformational processes of the data (in content analytic and artistic processes) have led to the emergence of the following categories: 1. Only me; 2. An impulse hits me/permeates me—I encounter myself; 3. Between outside and inside; 4. From inside to the outside; 5. Become quiet, listen carefully, watch, marvel; 6. A change. The outside rater proposed this sequential arrangement. We interpret the sequence as the temporal process of active creation that can contribute to theory development within and outside of the theory framework of embodied aesthetics [2]. Further results of the aesthetic transformations and synergizing performance are discussed in detail in Lange and Koch [27].

### 4.1. Limitations

We found full mediation for both outcomes *HSI* and *GSE* but need to point out that the small sample is at the lower limit of statistical power. In future studies the sample size would need to be increased. Also, the imbalance of women and men as well as the lack of random assignment are limitations of this study. It remains unclear, whether other factors not assessed here have a comparable effect on well-being and self-efficacy while and beyond creating: qualitative results, particularly the multitude of self-phrased expressions, capturing the individual experiences of the active person, suggest more working factors than the four items of the *ECS*. The fact that the ECS consists of only four items with two emerging dimensions (see Appendix B) causes reliability constraints. Now that the usefulness of the *ECS*-concepts for affecting psychological outcomes has been initially demonstrated, it would strengthen the measure to elaborate each of the two dimensions into five to six items and develop the scale into a ten to twelve item measure. Last but not least, it remains unknown whether the resulting factors are therapeutic factors since this research was done with a population of CAT students, and not with a clinical population. Clinical studies need to follow. 

Alternative explanations of results may have been the participants wanting to be “good participants” by providing social desirable answers (expectancy effects). Particular the well-being measure is quite transparent in study intent, particularly given the fact that we employed it directly before and after the intervention. Self-efficacy should be less subject to social desirability [24]. Another effect that may have caused increased well-being is the extra attention that the researcher provided to the participants, and that was not provided in a similar way to the control group, at least not in a 1:1 context (Hawthorn Effect). Future studies could focus on more concrete outcome variables that depend less on social desirability such as depression, anxiety, or self-esteem and are appropriate to the patient population under investigation.

**Figure 7:**
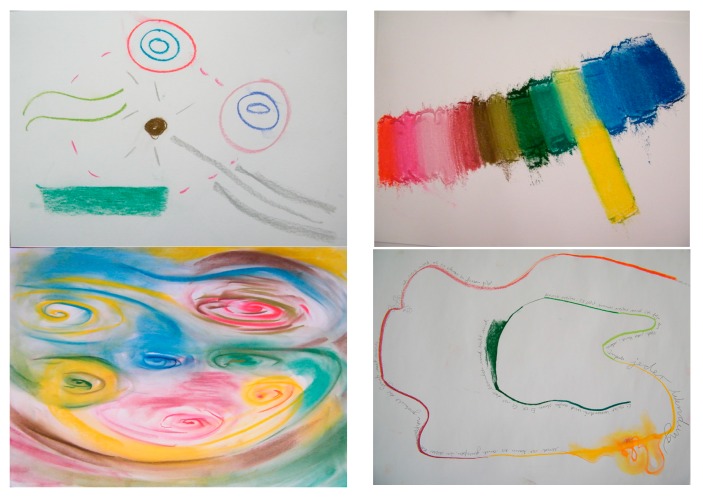
The third four examples of the creations with paper, color, and/or words that emerged from the artistic inquiry in this study.

**Figure 8:**
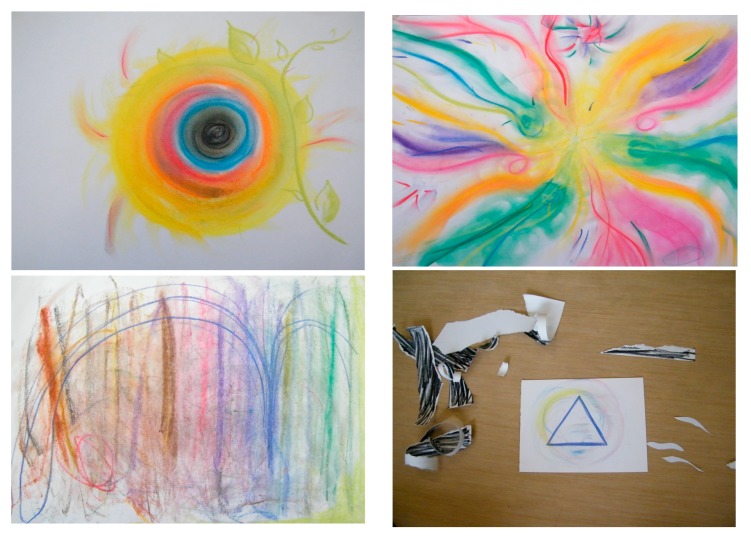
The last four examples of the creations with paper, color, and/or words that emerged from the artistic inquiry in this study.

Limitations on the qualitative side of data analysis is the subjectivity of parts of the data transformation processes (that on the other hand is purposefully employed as part of the method of artistic inquiry [20,27]; see Figure 3, Figure 4, Figure 7, and Figure 8 for illustrating examples of artistic inquiry). Future studies should identify points in the process, where the subjectivity can be controlled through measures of observer agreement and consider the determination of inter-rater-reliability as a possibility to establish higher objectivity, reliability, and validity of the results.

### 4.2. Future Research Considerations

Future research should be conducted with a bigger sample that is more balanced between male and female participants. In addition, participants should be assigned randomly to either experimental or control group. For better insight in creating in CATs, two more elements need further attention: this study should be conducted with a patient population in order to obtain results about clinical mechanisms. We assume that *ECS* is a therapeutic factor, since creating is a CAT intervention. This needs to be assessed.

Second, this study should be prolonged in time: creating should be operationalized as a process in time in which different emotional, cognitive, and intuitive shares get activated at different stages. Because of the restricted time frame of this study, creation had been operationalized as improvisation with artistic media by EG participants that had prior experience with improvising with artistic media. Also, the improvisation has been framed by the opening question: What happens inside yourself when you create? This was our attempt to grasp the entity of creating. In future research, the context in which creation takes place in CATs should be a clinical setting to increase external validity, which includes no condition of participants having prior experience with artistic media.

The Model of Embodied Aesthetics comes with loosely assigned common therapeutic factors [2] (p. 89). This feasibility study makes a proposal of how to go about the evidence-base of therapeutic factors in CATs: In the future, single CATs interventions should be assessed to find out about the specific and concrete cluster of fundamental mechanisms that then again can be fused into the Model of Embodied Aesthetics on an abstract level. The hypothesized factors can be based on the empirical data of qualitative and quantitative results. Furthermore, future research should address the missing procedural concept of therapeutic factors in CATs: Which factor comes in when and where, and how does the process differ depending on inter- and intrapersonal situations and circumstances?

Another important question of distinction needs to be asked with regard to active creation and activity. Does active creation have a positive effect on human health, because being active has a positive effect on human health? This question cannot be clarified with the results in this study, since active creation is a possibility of being active but should be taken into future research by implementing different control settings. 

Mediation analysis was used in order to evaluate quantitative data. The design and the modus operandi fit very well to the question of therapeutic factors in CATs. Hayes emphasizes that statistic constructions are models that we use for inference: “All we can do is use our data to try to model what that reality looks like” [19] (p. 53). We would like to encourage future researchers in this line of thought to employ the possibilities offered by mediation analysis with its parallel and serial models and moderation analysis in order to approximate the interrelations of the variables.

### 4.3. Implications for Practice

Since there are joint mechanisms of all arts therapies, it is important to investigate them closely and also to employ creative arts therapies more as a joint offer in clinical practice: the freedom of choice of the arts modality seemed to have been an important active factor for our students (Figure 3, Figure 4, Figure 7, and Figure 8 show examples of the chosen media colors and/or words) and can be assumed to be similarly important for the patients in our clinical institutions. For the patients, one of the working factors may precisely be the fact that he/she can chose the arts modality, he/she wants to express himself in. Freedom of choice increases one’s feeling of being taken seriously, and increases the experienced control, and thus the self-efficacy. 

Taking this set-up to the clinical context, one would need to make some relevant adaptations, the most important one being: to provide patients with more time and a broader introduction to the creative possibilities and materials. While our students were able to almost instantly immerse into creative activities across modalities, patients would need more security, explanation and time to get into the creative process (maybe initially a choice of two arts modalities would be enough for them, depending on the population). However, in the field of creative arts therapies there is much differentiated knowledge and daily experience on how to accompany patients into creative processes. The selected health-related psychological outcomes are central to psychological health and the chosen scales are suited for and tested with patients. As described above, the measures in our study are geared toward positive outcomes; in a similar study with patients, it would be advisable to additionally employ appropriate measures of symptom relief (e.g., regarding depression, anxiety, loneliness, or stress reduction). This study lays the groundwork for looking at more complex issues of psychological health paired with self-efficacy and well-being.

All in all, the chosen participatory approach is intended and suited to contribute to the empowerment of the patients, and is thus in line with a transformational approach of patient advocacy. 

## 5. Conclusions

The presented work is a feasibility study in the research field of therapeutic factors in CATs. The results are promising: The positive, indirect effect of active creation through *ECS* is statistically significant for both outcomes well-being (*HSI*) and self-efficacy (*GSE*); in addition, a high internal consistency of the *ECS* can be reported, with Cronbach’s Alpha of 0.89. At the same time, a first theoretical proposal is made about the nature of active factors and the relation of their concrete and abstract implementation in the therapeutic process. Thereupon, future research can build in order to gain insight into how exactly CATs work and where their overarching and specific factors lie.

## Figures and Tables

**Table 1:** Demographic data of participants.

Criterion		Total		EG		CG	
Numbers/percent		***N***	**%**	***N***	**%**	***N***	**%**
Participants		44	100	21	47.73	23	52.27
Gender	female	37	84.10	19	90.48	18	78.26
	male	7	15.90	2	9.52	5	21.74
Age	*M*	25.89		24.95		26.74	
	*SD*	6.67		6.53		6.81	
	min/max	19/52		19/44		20/52	
Subject/Major	DMT	13	29.54	9	42.86	4	17.39
	Music Therapy	24	54.55	5	23.81	19	82.61
	Occu. Th. (OT)	7	15.91	7	33.33		
Degree	BA	23	52.27	10	47.62	13	56.52
	MA	21	47.73	11	52.38	10	43.48
Previous experience with artistic media *		4.36		4.38		4.35	

Notes. DMT = dance movement therapy, OT = occupational therapy, EG = experimental group, CG = control group, *assessed with the use of a rating-scale where “1” means “no previous experience” and “5” means “maximum previous experience, *t*-test and Chi-square test yielded that there were no significant baseline differences between EG and CG (all *p*-values > 0.279).

**Table 2:** Means and standard deviations (SD) in experimental group (EG) and control group (CG) at both data collection times.

Outcome	Sample	*N*	*M*	*SD*
*HSI* (t1)	EG	19	4.07	0.60
	CG	22	3.92	0.59
	Total	41	3.99	0.59
*HSI* (t2)	EG	19	4.57 *	0.25
	CG	22	3.98 *	0.56
	Total	41	4.25	0.53
*GSE* (t1)	EG	22	3.01	0.32
	CG	22	3.06	0.44
	Total	44	3.04	0.39
*GSE* (t2)	EG	22	3.27 *	0.29
	CG	22	3.03 *	0.44
	Total	44	3.14	0.39
*ECS* (t1)	EG	22	3.95	0.52
	CG	22	3.99	0.42
	Total	44	3.97	0.46
*ECS* (t2)	EG	22	4.61 *	0.38
	CG	22	3.77 *	0.72
	Total	44	4.16	0.72

Note. EG = experimental group (*N* = 19), CG = control group (*N* = 22), total (*N* = 41), *HSI* = Heidelberg State Inventory [16], *GSE* = General Self-Efficacy Scale [24], *ECS* = Experience of Creation Scale, t = time of measurement; * *p* < 0.01 (group differences of EG and CG at posttest).

**Table 3:** Correlations of scales.

Outcome		*ECS* (t1)	*HSI* (t1)	*GSE* (t1)	*ECS* (t2)	*HSI* (t2)	*GSE* (t2)
*ECS* (t1)	Pearson’s *r*	1					
	*p*						
*HSI* (t1)	Pearson’s *r*	0.41 **	1				
	*p*	0.008					
*GSE* (t1)	Pearson’s *r*	0.35 *	0.30	1			
	*p*	0.026	0.058				
*ECS* (t2)	Pearson’s *r*	0.47 **	0.25	0.35 *	1		
	*p*	0.002	00.121	0.024			
*HSI* (t2)	Pearson’s *r*	0.37 *	0.63 **	0.42 **	0.69 **	1	
	*p*	0.017	0.000	0.007	0.000		
*GSE* (t2)	Pearson’s *r*	0.19	0.30	0.78 **	0.53 **	0.65 **	1
	*p*	0.236	0.060	<0.001	<0.001	<0.001	

Note. *N* = 41, *HSI* = Heidelberg State Inventory [16], *GSE* = General Self-Efficacy Scale [24], *ECS* = Experience of Creation Scale, *t* = time of measurement, ** *p* < 0.01, * *p* < 0.05.

**Table 4:** Item-scale statistics.

Item	Cronbach’s Alpha, When Leaving Item out	Cronbach’s Alpha with All 4 Items
Empowerment	0.875	0.886
Freedom	0.877	
Agency	0.815	
Creativity	0.840	

**Table 5:** Coefficients for the effect of active creation on well-being (*HSI*).

Antecedent	Consequent							
	*M* (*ECS*)				*Y* (*HSI*)			
		*Coefficient*	*SE*	*p*		*Coefficient*	*SE*	*p*
*X*	*A*	−0.839	0.186	0.000	*c*′	−0.209	0.177	0.243
*M* (*ECS*)					*b*	0.452	0.123	0.000
*Constant*	*i*(*a*)	5.451	0.296	0.000	*i*(*b*)	2.650	0.123	0.000
	*R*^2^ = 0.343				*R*^2^ = 0.444			
	*F*(1,39) = 20.367, *p* < 0.001				*F*(2,38) = 15.185, *p* < 0.001			

Note. *X* = Active creation, *ECS* = Experience of Creation Scale [26], *HSI* = Heidelberg State Inventory [16]; all variables included were taken from posttest (t2).

**Table 6:** Coefficients for the effect of active creation on self-efficacy (*GSE*).

Antecedent	Consequent							
	*M* (*ECS*)				*Y* (*GSE*)			
		*Coefficient*	*SE*	*p*		*Coefficient*	*SE*	*p*
*X*	*A*	−0.856	0.176	0.000	*c′*	0.057	0.137	0.680
*M* (*ECS*)					*b*	0.332	0.096	0.001
*Constant*	*i_a_*	5.451	0.282	0.000	*i*	1.635	0.552	0.005
	*R^2^* = 0.361				*R^2^* = 0.285			
	*F*(1,42) = 23.722, *p* < 0.001				*F*(2,41) = 8.171, *p* = 0.001			

Note. *X* = Active creation, *ECS* = Experience of Creation Scale, *GSE* = General Self-Efficacy Scale [24].

## Appendix A

**Table A1:** Heidelberg State Inventory (*HSI*; English Translation; SK).

I Momentarily Feel	
Item	1 = Not at All True 5 = Exactly True
Relaxed	1 2 3 4 5
In a good mood	1 2 3 4 5
Full of resources	1 2 3 4 5
Without worries	1 2 3 4 5
Indulgent	1 2 3 4 5
Tense	1 2 3 4 5
In a bad mood	1 2 3 4 5
Depressed	1 2 3 4 5
Anxious	1 2 3 4 5
Empty, without life	1 2 3 4 5
Ready to deal with the world	1 2 3 4 5
Full of energy	1 2 3 4 5
Positive	1 2 3 4 5
Strong	1 2 3 4 5
Fighting	1 2 3 4 5
Without resources	1 2 3 4 5
Negative	1 2 3 4 5
Full of worries	1 2 3 4 5
Not ready to deal with theworld	1 2 3 4 5
Without energy	1 2 3 4 5
Without strength	1 2 3 4 5
Not depressed	1 2 3 4 5
Not anxious	1 2 3 4 5
Full of life	1 2 3 4 5

Note: English version was first published in bipolar format [15], and in unipolar format [16].

## Appendix B

**Table A2:** Experience of Creation Scale (*ECS*; English Translation; GL).

I Momentarily Feel	
Item	1 = Not at All True 5 = Exactly True
Empowered *	1 2 3 4 5
Free to decide **	1 2 3 4 5
Effective/That I have an impact ***	1 2 3 4 5
Creative ****	1 2 3 4 5

Note. Original German words: * selbstermächtigt, befähigt, potent; ** frei zu entscheiden; *** so, dass ich was bewirken kann; **** kreativ; within the four items freedom and creativity were stronger related, as well as empowerment and impact.
